# The extreme capsule and aphasia: proof-of-concept of a new way relating structure to neurological symptoms

**DOI:** 10.1093/braincomms/fcab040

**Published:** 2021-03-14

**Authors:** Ariane Martinez Oeckel, Michel Rijntjes, Volkmar Glauche, Dorothee Kümmerer, Christoph P Kaller, Karl Egger, Cornelius Weiller

**Affiliations:** Department of Neurology and Clinical Neurosciences, Faculty of Medicine, University of Freiburg, Freiburg 79106, Germany; Department of Neurology and Clinical Neurosciences, Faculty of Medicine, University of Freiburg, Freiburg 79106, Germany; Department of Neurology and Clinical Neurosciences, Faculty of Medicine, University of Freiburg, Freiburg 79106, Germany; Department of Neurology and Clinical Neurosciences, Faculty of Medicine, University of Freiburg, Freiburg 79106, Germany; Department of Neuroradiology, Faculty of Medicine, University of Freiburg, Freiburg 79106, Germany; Department of Neuroradiology, Faculty of Medicine, University of Freiburg, Freiburg 79106, Germany; Department of Neurology and Clinical Neurosciences, Faculty of Medicine, University of Freiburg, Freiburg 79106, Germany

**Keywords:** aphasia, stroke, dual pathway model, extreme capsule, arcuate fascicle

## Abstract

We present anatomy-based symptom-lesion mapping to assess the association between lesions of tracts in the extreme capsule and aphasia. The study cohort consisted of 123 patients with acute left-hemispheric stroke without a lesion of language-related cortical areas of the Stanford atlas of functional regions of interest. On templates generated through global fibre tractography, lesions of the extreme capsule and of the arcuate fascicle were quantified and correlated with the occurrence of aphasia (*n*** **=** **18) as defined by the Token Test. More than 15% damage of the slice plane through the extreme capsule was a strong independent predictor of aphasia in stroke patients, odds ratio 16.37, 95% confidence interval: 3.11–86.16, *P*** **<** **0.01. In contrast, stroke lesions of >15% in the arcuate fascicle were not associated with aphasia. Our results support the relevance of a ventral pathway in the language network running through the extreme capsule.

## Teaser

**Abbreviated summary**

Martinez Oeckel et al. report that aphasia after stroke is associated with lesions of the extreme capsule fibres and not with lesions of the arcuate fascicle. They use a new promising method of anatomy-based lesion-symptom mapping, mapping lesions of either tract on the occurrence of aphasia in acute stroke patients.

## Graphical Abstract

**Graphical Abstract:**
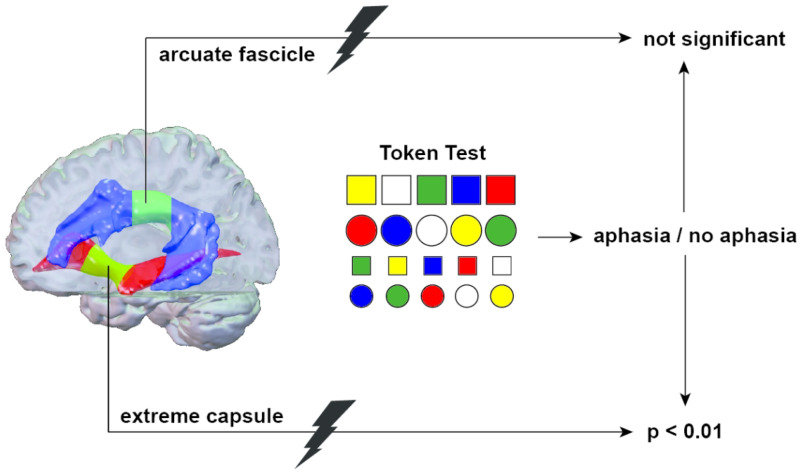

## Introduction

Relating brain anatomy and neurological symptoms has gained the interest of researchers since the 19th century. Correlating a pathological phenotype to anatomically defined lesions such as in stroke is the oldest approach in this field, first done in post-mortem brains,^1^ and meanwhile made possible in vivo by the advent of imaging techniques. Today, its voxel-based variant using magnetic resonance imaging (MRI) to map symptoms onto lesions is a driving force in human brain mapping and thought to identify causal relationships between brain and behaviour.^2^ However, the established univariate voxel-based lesion-symptom mapping (VLSM) method^3^ has been challenged because of its topographical bias, resulting in a misplacement towards the centre of major arteries.^4^^,^^5^

The reverse approach, choosing a defined anatomical structure and relating its affection to the occurrence of symptoms, may be called ‘anatomy-based symptom-lesion mapping’ (ASLM) in analogy to VLSM. ASLM has rarely been used and calls for a different procedure than VLSM. ASLM requires several a-priori conditions to achieve a high specificity: (i) a brain function linked to an established ‘anatomical network’ and corresponding neurological syndrome, (ii) within this network a defined anatomical structure of interest and techniques to unambiguously identify this anatomical structure and (iii) the selection of patients without lesions in other parts of the anatomical network to ensure that the clinical syndrome is due to the selected region. Thus, while VLSM relates scores or binary behavioural data to lesions on a voxel-by-voxel basis, ASLM selects an anatomically defined structure and relates its lesioning to the occurrence of a symptom or a syndrome while carefully avoiding affection of other parts of the network. There are other studies relating lesioning of tracts to the occurrence of symptoms*.*^6^ We are not aware of a comparable publication following these preconditions vigorously and present a proof-of-concept study to investigate how such a technique can be useful. The three preconditions for our study are explained in the following: (i) Language is a well-studied brain function with a corresponding classical syndrome, aphasia, which is assessed by validated clinical tests. Language involves different domains and overlaps with other cognitive systems that process motor function, sensation, emotion, memory and others^7^^,^^8^ and a widespread, bilateral network with many cortical and subcortical areas is involved in language processing.^9^ An atlas that comprises these cortical language-relevant regions is the Stanford atlas.^10^ (ii) The most prominent functio-anatomical model for language processing is the dual-stream language network.^11^^,^^12^ In this model, several temporal areas and inferior parietal cortex are connected with the ventrolateral frontal region and lateral premotor cortex along two major associations tracts.^13^^,^^14^ The ‘dorsal pathway’ in this model is constituted by the arcuate fascicle (AF; syn. fasciculus arcuatus^15^), traditionally been considered as the most important tract in language system processing.^16^^,^^17^ In the ventral pathway, the situation is less clear. The most prominent ventral tracts, which continue to the frontal cortex are the inferior frontal-occipital fascicle and the uncinate fascicle. The inferior frontal-occipital fascicle by definition does not connect temporal regions,^18^ the uncinate fascicle may be more related to abstract representations or is seen as part of the limbic system.^19^ A direct ventral temporo-frontal connection between what may be seen as Wernicke’s and Broca’s areas in humans has unequivocally been demonstrated in nonhuman primates using autoradiographic tracing and termed ‘extreme capsule fascicle’ (ECF).^14^^,^^20^ Similar fibres in the extreme capsule have been identified *in vivo* in humans using Diffusion Tensor Imaging (DTI)-based fibre tracking^21^^,^^22^ and stimulated a ‘rethinking of the language circuity’.^23^ In contrast to the AF, the function of the ventral connection to the frontal lobe for language processing and its importance in the development of aphasia is less obvious.^17^^,^^24^^,^^25^ Thus, we decided to study the ECF within the anatomical region ‘extreme capsule’. (3) ASLM requires the exclusion of patients with lesions to the functional network other than the anatomical structure under study, here the ECF. We decided to exclude patients with lesions affecting the language-relevant cortical regions of interests (ROIs) as defined by the Stanford atlas language network^10^ and took the AF as control area.

We explored the anatomical region ‘extreme capsule’ and its relation to the syndrome ‘aphasia’ as tested by the Aachen Aphasia Test (AAT),^26^ the most widely used aphasia test in German, in patients with acute (<10** **days) first ever stroke due to a singular ischaemic infarct of the left hemisphere. We asked the simple question whether a lesion of the extreme capsule leads to aphasia, taking the AF as control structure.

## Materials and methods

**Figure 1:**
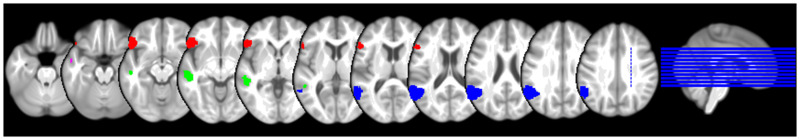
**Location of language zones.** Four left-hemispheric functional regions of interest of the Stanford atlas language network (red: inferior frontal gyrus; lilac: anterior temporal lobe; green: middle temporal gyrus; blue: superior temporal gyrus), modified from Shirer et al.^10^^,^^30^

The study was approved by the local ethics committee of the University Medical Centre of Freiburg (281/13). Patients were recruited from the Stroke Unit or the intensive care unit, Department of Neurology at the University Medical Centre of Freiburg, Germany, in the context of a large prospective study (‘Freiburg large scale project’) on stroke-related cognitive deficits.^27–29^ All patients with acute stroke consecutively admitted to the Stroke Unit or intensive care unit were screened. As ischaemic infarcts and intracranial bleedings have different pathophysiology, different anatomical locations, and different mechanisms to produce symptoms, we decided to only study acute ischaemic infarcts within 10** **days and exclude hemodynamic infarcts. The inclusion criteria were (i) embolic first-time stroke of the left middle cerebral artery-territory, (ii) age < 90** **years and (iii) German native speaker. Exclusion criteria included (i) recurrent stroke during the study period, (ii) previous stroke, previous intracerebral bleeding, previous traumatic brain injury, (iii) inability to tolerate MRI examination or clinical testing due to reduced general health status, (iv) hearing or visual deficits, (v) cognitive impairment other than aphasia and (vi) any contraindication to MRI. In the period between February 2011 and December 2018, we screened 7405 patients; 287 with acute ischaemic left-hemispheric stroke met the inclusion and exclusion criteria. In five patients, aphasia testing was not possible within 10** **days after stroke. To further restrict the 282 patients to those with infarct areas outside the cortical language-zones, overlaps between stroke lesion and the four left-hemispheric functional ROIs of the Stanford atlas language network (Fig. 1) were calculated and all patients with lesions (>0%) in at least one of the four ROIs were excluded (*n*** **=** **150).

The study outcome was aphasia as a binary variable. The Token Test was found to discriminate particularly well between patients with aphasia and normal controls,^31^ normal hospitalized adults,^32^ non-aphasic right-hemisphere-damaged adults^33^^,^^34^ and non-aphasic diffuse- and focal-brain-damaged adults.^35^ To assess aphasic deficits in our cohort, all patients completed the Token Test of the AAT.^26^ Aphasia was diagnosed for patients with deficits of at least seven age-corrected error scores according to the allocation of the AAT. In nine patients, categorization was ambiguous. These patients were allocated to the non-aphasia group as they scored <7 error points in the Token Test; however, they scored <78 of 90 points in the AAT subtests for reading and writing. We decided to exclude these nine patients from the study to ensure unharmed language function in the non-aphasia group.

Full written consent was obtained from all subjects. In cases of severe aphasia or paralysis of the right hand, detailed information was given to the patient’s relatives or the legal guardian. The study was approved by the local ethics committee.

MRI acquisition: on a 3-T TIM TRIO scanner with a 32-channel head coil (Siemens, Erlangen, Germany) diffusion-sensitive single-shot spin-echo echo-planar imaging sequence was acquired with the following parameters: 61 diffusion encoding gradient directions (b-factor, 1000** **s/mm^2^); repetition time, 11 800** **ms; echo time, 96** **ms; inversion time, 2300** **ms; 69 axial slices; matrix size, 104 × 104; field of view, 208** **mm; voxel size, 2 mm × 2 mm × 2 mm. Nine additional scans without diffusion weighting (b-factor, 0** **s/mm^2^) were equally distributed across the acquisition series, resulting in a total of 70 volumes. For anatomical correlation T_1_-weighted magnetization-prepared rapid gradient echo sequence was acquired with the following parameters: repetition time, 2200** **ms; echo time, 4.11** **ms; inversion time, 1100** **ms; flip angle, 12°; 160 sagittal slices; matrix size, 256 × 256; field of view, 256** **mm; voxel size, 1 × 1 × 1 mm^3^.

DTI processing: DTI data were processed using a MATLAB-based in-house toolbox for fibre tracking.^36^ Parameters for global tracking were a cylinder width of 1** **mm and a cylinder length of 3** **mm. The weight of a cylinder segment was set to one-fourth of the brain-averaged anisotropic signal component, resulting in a ‘dense’ reconstruction with an average of 30 cylinders per voxel. Note that the weight parameter is comparable to a fractional anisotropy threshold: for higher weights, the number of streamlines is reduced and a significant amount of streamlines is only revealed for regions with a highly anisotropic diffusion distributions. Conversely, a lower weight leads to high number of streamlines, even in regions with a low fractional anisotropy. Given the low weight selected here, the number of iterations was set to 3 × 10^8^. Finally, the temperature schedule for the cooling phase of the polymerization process was chosen exponentially with a starting temperature of 0.1 to a stop temperature of 0.001.^37^

**Figure 2:**
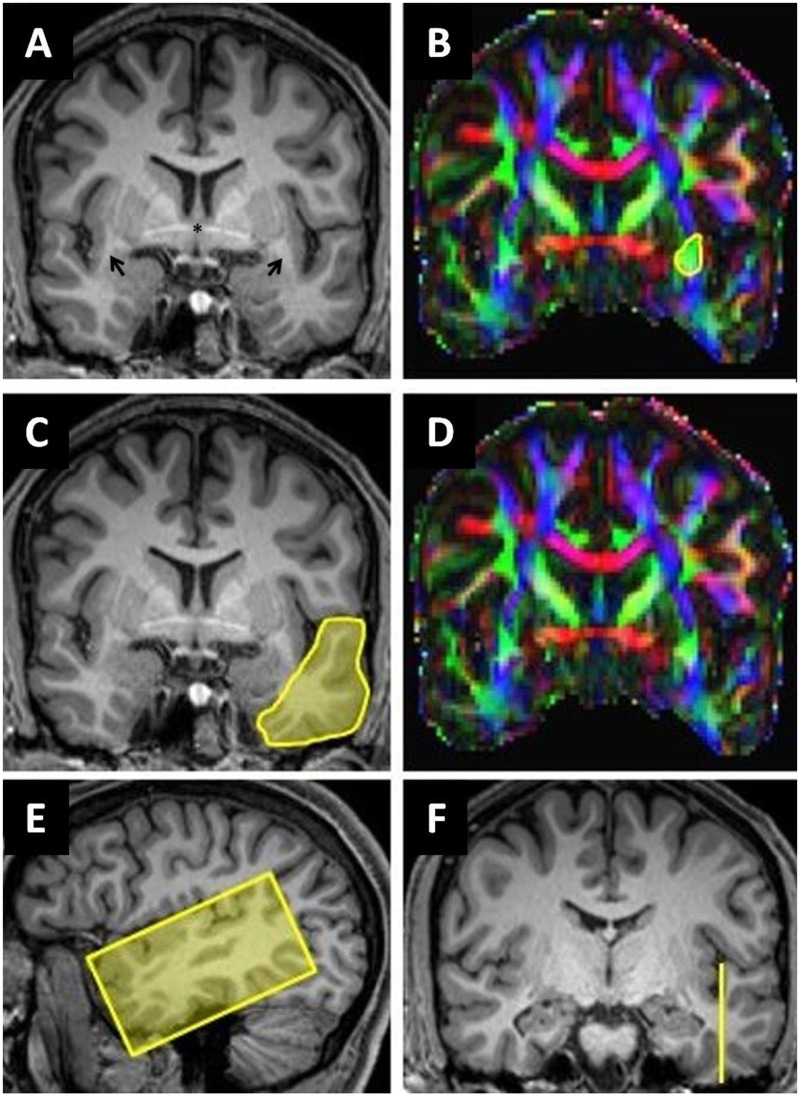
**Segmentation of extreme capsule streamlines.** (**A**, **C** and **F**) Coronal reformatted MPRAGE (magnetization-prepared rapid acquisition with gradient echo) images. (**E**) Sagittal reformatted MPRAGE image. (**B** and **D**) Corresponding colour-coded fractional anisotropy maps: the arrows in **A** show the location of the temporal stem in both hemispheres. The yellow circle in **B** (ROI1) includes all green voxels in anterior extreme capsule/temporal stem at the level of the anterior commissure including extreme capsule streamlines. The yellow area in **C** (ROI2) covers the entire cross-section of the anterior temporal lobe. ROI3 (**E** and **F**) was placed in a sagittal plane barely cutting the ground of the temporal sulcus to segment cortical projection streamlines of the temporal lobe.

**Figure 3:**
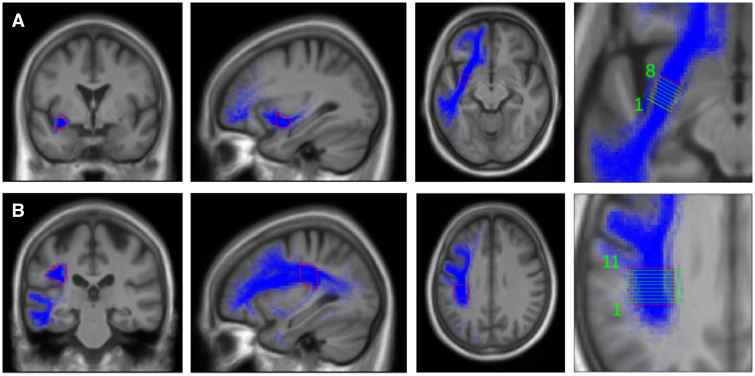
**Extreme capsule fibre template and arcuate fascicle template.** Regions of interest and slices (perpendicular to the main fibre orientation) in the extreme capsule fibre system (**A**) and arcuate fascicle fibre system (**B**). Coronal, sagittal and horizontal planes of templates. Fibre system (blue), outline of template (red), layers in template (green).

Bundle selection: we used the imaging and tracking methodology and selection of AF streamlines as described previously.^38^ Detailed description of the ROIs used for the ECF streamline selection are shown in Fig. 2. First ROI1 and ROI3 were used to include (‘and’ function) from the temporo-lateral region running through the anterior part of the extreme capsule/external capsule, secondary ROI2 was used to exclude streamlines (‘not’ function) from the temporo-polar region. To determine the amount of disruption of the fibre bundles, we selected the mid portion of the bundles, where most of the fibres can be assumed to run through a very circumscribed region. These regions were cut into slices perpendicular to the main fibre orientation. Figure 3A shows the ROIs and slices in the ECF, Fig. 3B in the AF. For each slice of these ROIs, we calculated the percentage of overlap between the fibre bundle area in that slice and the lesion of each patient. Assuming that association fibres are discontinued as soon as the lesion overlaps with at least one slice of an ROI, we computed the maximum percentage of overlap across the slices of ECF and AF ROIs for each patient.

Stroke volume, age and gender were covariates. Total normalized stroke volume was measured voxel-based on diffusion-weighted MRI data and indicated in millilitres. Patients were dichotomized in those with or without aphasia mainly by means of the internationally established Token Test.^39^ Patients were selected that infarcts did not affect the language-relevant cortical ROIs as defined by the Stanford atlas language network.^10^^,^^30^ The association between ECF lesions and aphasia was calculated with Fisher’s exact test. In the absence of an established parameter for lesions of tracts, we dichotomized ECF lesions arbitrarily into two equally sized groups using the statistical parameter median split. We further split the group with ECF lesions ≥15% into subgroups using the upper quartile threshold of 30%. The reference group consisted of stroke patients with no lesion of the ECF (0%). Patients with AF lesions were dichotomized accordingly by using the median split. We used multivariate logistic regression to estimate the odds ratio (OR) of aphasia, associated with ECF lesions compared with no ECF lesions, adjusting for AF lesions, stroke volume as a continuous variable, age (dichotomous with cut-off at 70** **years) and gender.

### Data availability

The data that support the findings of this study are available from the corresponding author upon reasonable request.

## Results

The study cohort consisted of 123 patients with acute left-hemispheric ischaemic infarcts without lesions in the functional ROIs of the language network. Testing for aphasia was performed within 10** **days after their first-time stroke (mean 3.93 ± 2.28** **days, range 0–10** **days). Aphasia was diagnosed in 18 patients with >7 error scores in the Token Test, 105 were qualified as having no aphasia in the Token Test as well as in the subtest reading and writing of the AAT. The mean age was 64.5 ± 14.5** **years and 63.4% were men. ECF lesions were present in 24.4% (30 of 123 patients), and AF lesions in 37.4% (46 of 123 patients), including 13.8% (17 of 123 patients, 5 of whom with aphasia and 12 without aphasia) with simultaneous ECF lesions. The median split of both ECF and AF lesions was 15%. The mean stroke volume was 6.9 ± 8.0 ml, 6.0 ± 7.4 ml in those without and 11.7 ± 10.0 ml in those with aphasia. Aphasia occurred in 14.6% (18 of 123 patients) (Table 1). There was no difference in the intervals between stroke onset performance of Token Test or MRI scanning between both groups. Individual data of all aphasia patients are shown in Table 2.

**Table 1:** Demographics of cohort with ischaemic left-hemispheric stroke

	Without aphasia	With aphasia	All patients	*P*-value^c^
Total	*N* = 105	*N* = 18	*N* = 123	
Gender				
Male	66/105 (62.9)	12/18 (66.7)	78/123 (63.4)	1.00
Female	39/105 (37.1)	6/18 (33.3)	45/123 (36.6)	
Age (years)				
Mean (SD)	63.4 (14.5)	70.7 (13.3)	64.5 (14.5)	<0.05
Median (p25, p75)	65/105 (57, 75)	73 (70, 81)	67 (57, 76)	
<70	62/105 (59.0)	5/18 (27.8)	67/123 (54.5)	
≥70	43/105 (41.0)	13/18 (72.2)	56/123 (45.5)	
Stroke volume (ml)				
Mean (SD)	6.0 (7.4)	11.7 (10.0)	6.9 (8.0)	<0.05
ECF lesion^a^^,d^				
None	85/105 (81.0)	8/18 (44.4)	93/123 (75.6)	<0.05
<15%	15/105 (14.3)	1/18 (5.6)	16/123 (13.0)	
≥15%	5/105 (4.8)	9/18 (50.0)	14/123 (11.4)	
Mean (SD)^b^	19.3 (24.9)	37.3 (21.7)	25.3 (25.0)	<0.05
AF lesion^a^^,d^				
None	65/105 (61.9)	12/18 (66.7)	77/123 (62.6)	0.80
<15%	20/105 (19.0)	3/18 (16.7)	23/123 (18.7)	
≥15%	20/105 (19.0)	3/18 (16.7)	23/123 (18.7)	
Mean (SD)^b^	20.6 (21.5)	20.4 (17.5)	20.6 (20.9)	0.80

p = percentile; SD = standard deviation.

a Lesions dichotomized according to their median split.

b Of those with a lesion.

c Comparing patients with and without aphasia using Fisher’s exact test for categorical variables and Student’s *t*-test for continuous variables.

d Including 17 patients with simultaneous ECF and AF lesions, 5 of whom with aphasia and 12 without aphasia.

**Table 2:** Individual data of all aphasia patients

Patient	Token Test error points^a^ (percentile rank)	Token Test subtest score of total (%)					AAT subtests percentile rank				ECF lesion^b^	AF lesion^b^	Lesion site
		1	2	3	4	5	Written language	Repetition	Naming	Compre hension			
1	9 (83)	100	90	80	40	40	65	70	59	65	31	41	Basal ganglia
2	11 (79)	100	90	80	30	30	97	92	94	55	–	–	Thalamocapsular area
3	10 (81)	100	70	70	50	50	95	84	91	94	–	–	Frontoopercular area
4	9 (83)	100	90	80	20	60	72	83	44	25	19	2	Striatocapsular area
5	50 (2)	0	0	0	0	0	5	2	6	2	70	–	Lateral sulcus
6	36 (36)	70	20	0	0	0	16	60	16	3	–	–	Frontoinsular area
7	7 (89)	90	80	80	60	60	60	77	60	53	–	–	Precentral area
8	10 (81)	80	100	80	30	50	70	89	99	76	–	13	Corona radiata
9	9 (83)	100	90	70	30	60	99	99	99	70	50	24	Basal ganglia
10	10 (81)	100	100	90	70	40	84	83	77	88	65	–	Basal ganglia
11	11 (79)	100	90	80	50	70	99	72	82	79	20	40	Striatocapsular area
12	18 (68)	100	80	50	0	30	73	97	62	70	21	–	Lenticulostriatal area
13	11 (79)	100	100	60	70	0	41	68	64	33	11	–	Basal ganglia
14	21 (63)	70	70	50	0	40	79	72	66	39	27	–	Lenticulostriatal area
15	38 (31)	30	20	10	0	0	11	22	14	11	59	2	Basal ganglia
16	13 (76)	90	100	50	50	50	66	94	98	59	–	–	Inferior frontal gyrus
17^c^	10 (81)	100	80	90	40	30	70	94	94	–	–	–	Semioval centre
18	7 (89)	100	90	50	70	60	100	92	98	100	–	–	Precentral area
Mean	70	85	76	59	34	37	67	75	68	54	–	–	–
SD	23	28	30	29	26	23	30	26	31	31	–	–	–

SD = standard deviation.

a Of a total of 50 points.

b Maximum percentage of overlap across the slices of the ECF and AF region of interest.

c Patient was unable to perform the comprehension subtest due to a recurrent stroke.

**Figure 4:**
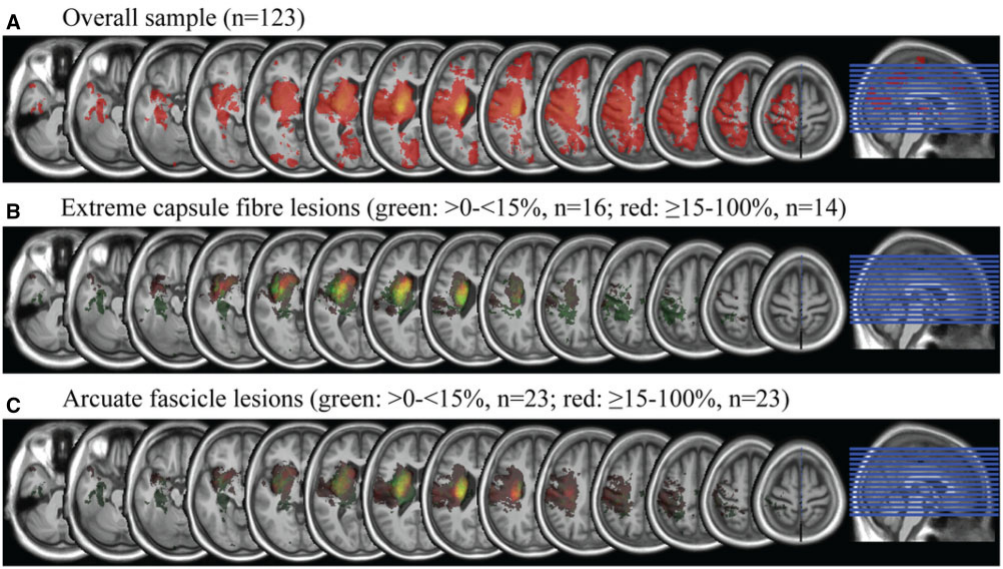
**Lesion overlap of all patients and subgroups with extreme capsule fibre and arcuate fascicle lesions.** Equidistant slices from *z* = −33 to *z* = 64.

Aphasia was significantly more frequent in patients with lesions of the ECF (*P*** **<** **0.05), but not with lesions of the AF (*P*** **=** **0.80). The multivariate logistic regression analysis showed that stroke patients with >15% ECF lesion were at greater odds to be affected with aphasia than those without ECF lesions (OR** **=** **16.37, 95% confidence interval: 3.11–86.16, *P*** **<** **0.01). The subgroups with 15–30% ECF lesion (OR** **=** **18.31, 95% confidence interval: 1.86–180.77, *P*** **=** **0.013) as well as patients with >30% ECF lesion (OR** **=** **15.32, 95% confidence interval: 2.31–101.57, *P*** **=** **0.005) showed the same risk of aphasia. AF lesions of more than 15% were not associated with aphasia (OR** **=** **0.30, 95% confidence interval: 0.04–2.35, *P*** **=** **0.25) (Table 3). There was no interaction between ECF and AF lesions (*P*** **=** **0.71). Lesion overlaps of all 123 patients are displayed in Fig. 4.

**Table 3:** Predictors of aphasia in 123 patients with left-hemispheric stroke

	With aphasia, *n* (%)	Without aphasia, *n* (%)	Crude OR (0.95-CI)	Adjusted OR^a^ (0.95-CI)	*P*-Value^b^
Total	18 (100.0)	105 (100.0)			
ECF lesion^c^					
None	8 (44.44)	85 (80.95)	1	1	–
<15%	1 (5.56)	15 (14.29)	0.71 (0.08–6.08)	0.72 (0.07–7.08)	0.78
≥15%	9 (50.00)	5 (4.76)	19.12 (5.15–71.00)	16.37 (3.11–86.16)	<0.01
≥15% < 30%	4 (22.22)	2 (1.90)	21.25 (3.36–134.56)	18.31 (1.86–180.77)	0.01
>30%	5 (27.78)	3 (2.86)	17.71 (3.56–88.10)	15.32 (2.31–101.57)	<0.01
AF lesion^c^					
one	12 (66.67)	65 (61.90)	1	1	–
15%	3 (16.67)	20 (19.05)	0.81 (0.21–3.17)	0.33 (0.05–2.25)	0.26
≥15%	3 (16.67)	20 (19.05)	0.81 (0.21–3.17)	0.30 (0.04–2.35)	0.25
Other factors					
Female gender	6 (33.33)	39 (37.14)	0.85 (0.29–2.43)	0.70 (0.19–2.56)	0.59
Age ≥70** **years	13 (72.22)	43 (40.95)	3.75 (1.25–11.29)	3.97 (1.02–15.46)	0.05
Stroke volume (ml)^d^	11.68	6.04	1.07 (1.01–1.12)	1.06 (0.98–1.14)	0.13

CI: confidence interval; OR: odds ratio.

a Adjusted for ECF lesion, AF lesion, gender, age and stroke volume.

b Two-sided Wald-test derived from logistic regression.

c Lesions dichotomized according to their median split.

d Mean volume in ml presented instead of *n* (%) and used as continuous variable in the logistic regression models.

## Discussion

We present a proof-of-concept study relating the lesion of an anatomically defined structure to the occurrence of a neurological syndrome. Our findings show a significant association between lesions in the extreme capsule and aphasia in patients with acute stroke without cortical infarction of the language network regions. Patients with lesions of >15% of the slice plane of the ECF tract have an over 16 times higher OR of presenting with aphasia after stroke than patients without a lesion in this area, irrespective of any lesion of the AF. Lesions in the AF were not associated with aphasia as defined by the Token Test. Stroke volume, gender and age were not independently associated with a higher risk of aphasia.

This study introduces a new technique we name ASLM. ASLM is based on defining a brain ROI, in this study an association tract, and correlating it with behavioural dysfunction, here the presence of aphasia, in patients with intact cortical language processing regions. We are not aware of another study adopting this approach. There are VLSM studies relating aphasic symptoms to lesions of tracts.^24^^,^^25^^,^^28^^,^^40^ Others assess white matter integrity of established language association tracts outside the stroke lesion itself without controlling for involvement of the cortical language zones.

We found a strong and statistically significant association between ECF lesion and aphasia. There is an ongoing discussion about the correct naming of the language-related ventral tracts running through the extreme capsule, consisting of uncinate fascicle, inferior frontal-occipital fascicle and ECF.^14^^,^^41^ We took great care to separate the ECF but are well aware that the three tracts anatomically overlap within the extreme capsule,^42^ and thus may be not really differentiable conceptually. Thus, we studied the bulk of fibres running through the extreme capsule and each of the tracts may be associated with aphasia, not that lesions of either ECF, inferior frontal-occipital fascicle or uncinate fascicle are mainly responsible. Investigating the ECF lesions by using a DTI fibre tracking template and calculating the damage of plane slice in percent has enabled us to include qualitative and quantitative information on the damage of the ventral pathway. In the absence of an established parameter for defining ECF lesion cut-off, we arbitrarily set it at 15% using the median split. By further splitting the group with ECF lesions ≥15% into subgroups using the upper quartile threshold of 30%, we showed that the selection of this cut-off did not change the main study finding (Table 2).

We investigated acute stroke patients within 10** **days without any other pathology on MRI to ensure that clinical testing was not influenced by reorganization of the brain*.*^43^^,^^44^ We specified language regions with the Stanford atlas of functional ROIs and only included patients whose lesions did not overlap with such defined regions. The vigorous selection of patients increases specificity, the downside is the need for relatively large number of patients to select from. The size of the study cohort of 123 stroke patients including 18 (14.6%) with aphasia may be considered as a study limitation. However, we found a strong and statistically significant association between ECF and aphasia. The external validity of this strong association has to be proven in further studies. A larger study cohort with more aphasia cases could have yielded more precise OR estimates. Multicentre studies or cloud-based stroke depositories may present a solution to increase study size.

The Stanford atlas does not include all brain regions potentially involved in language processing. Consequently, we did not examine and can therefore not exclude that patients with ≥15% affection of the extreme capsule cluster have lesions of other regions that contribute to aphasia.

The Token Test of the German version of the AAT^26^ is generally accepted as a reliable instrument for distinguishing between aphasia and no aphasia.^45^^,^^46^ In this study, we defined aphasia by an impaired Token Test performance only. To guarantee a comparison group definitely without language problems we excluded patients of the non-aphasia group as defined by Token Test criteria, who, however, were affected in the subtest for reading and writing. Thus, both groups, aphasia and no aphasia, are reliably separated. We considered aphasia as dichotomous variable and did not specify subgroups.

The neuroscientific question we asked, does lesioning of the ventral language pathway lead to aphasia independent from cortical lesions or lesions of the AF can clearly be answered with yes. While language is mainly processed in the cortex,^9^ aphasia after subcortical pathology is well studied.^47^^,^^48^ Various mechanisms are discussed as participation of subcortical structures in language processing beyond articulation, diaschisis, cortical hypoperfusion with undetected affection of cortical neurons on imaging or disconnection phenomena.^49–55^

For decades, the AF was regarded as the major fibre tract for language processing connecting Broca’s with Wernicke’s area^16^^,^^17^ and reports about lesions of AF leading to aphasia are frequent.^56^^,^^57^ The discovery of a ventral pathway in the auditory system^58^ and tracing experiments in primates^20^ led to the formulation of a dual-stream model for language processing.^11^^,^^12^^,^^59^^,^^60^ The model is similar to the original formulation of a ‘direct’ and an ‘indirect’ pathway by Wernicke *(*although he correlated the ‘direct’ pathway anatomically with extreme capsule fibres^17^) and re-discovered with today’s DTI techniques.^22^ In the dual-loop model of language processing, temporal lobe, including Wernicke’s area, and inferior frontal cortex, including Broca’s area, are connected via two association tracts, allowing processing of language along two pathways with different computational abilities.^61^ A dorsal pathway through the AF and the superior longitudinal fascicle fibre system is used for sensorimotor mapping for correct speech production (‘mapping sound onto articulation’), storage and retrieval of sequences, whereas a ventral pathway running through the extreme capsule mainly serves ‘mapping sound onto meaning’.^8^^,^^11^^,^^13^^,^^21^^,^^22^^,^^62^ The ventral stream allows categorization of items based on their structure,^17^^,^^63^ which is just what the Token Test tests.^64^

Disruptive intraoperative electrostimulation of the ECF leads to semantic paraphasia.^65^ Damage of the ventral pathway is known to cause a deficit in language comprehension, while damage of the dorsal pathway impairs speech production.^28^^,^^66^^,^^67^ Thus, while the dorsal pathway is needed for correct speech production as well as processing of chunks,^68^ the ventral pathway is necessary for comprehension. The Token Test examines language comprehension in addition to attention and other cognitive functions, not however overt language production.^64^ The Token Test may thus be especially suited to test ‘ventral’ stream functions.

## Conclusions

The ASLM is a new and promising method. Our results support the relevance of a ventral pathway in the language network running through the extreme capsule. Extreme capsule lesions are an independent and strong predictor for aphasia after acute stroke, other than lesions in the AF.

## Abbreviations

AAT =: Aachen Aphasia Test
AF =: arcuate fascicle
ASLM =: anatomy-based symptom-lesion mapping
DTI =: Diffusion Tensor Imaging
ECF =: extreme capsule fibre
MRI =: magnetic resonance imaging
OR =: odds ratio
ROI =: region of interest
VLSM =: voxel-based lesion-symptom mapping
